# The molecular basis of differential morphology and bleaching thresholds in two morphs of the coral *Pocillopora acuta*

**DOI:** 10.1038/s41598-017-10560-2

**Published:** 2017-08-30

**Authors:** Hillary Smith, Hannah Epstein, Gergely Torda

**Affiliations:** 1 ARC Centre of Excellence for Coral Reef Studies, James Cook University, Townsville, Queensland 4811 Australia; 2 Australian Institute of Marine Science, PMB 3, Townsville, Queensland 4810 Australia; 3 College of Science and Engineering, James Cook University, Townsville, Queensland 4811 Australia; 4 AIMS@JCU, James Cook University, Townsville, Queensland 4811 Australia

## Abstract

Processes of cnidarian evolution, including hybridization and phenotypic plasticity, have complicated the clear diagnosis of species boundaries within the phylum. *Pocillopora acuta*, a species of scleractinian coral that was recently split from the widespread *Pocillopora damicornis* species complex, occurs in at least two distinct morphs on the Great Barrier Reef. Contrasting morphology combined with evidence of differential bleaching thresholds among sympatrically distributed colonies suggest that the taxonomy of this recently described species is not fully resolved and may represent its own species complex. To examine the basis of sympatric differentiation between the two morphs, we combined analyses of micro- and macro-skeletal morphology with genome wide sequencing of the coral host, as well as ITS2 genotyping of the associated *Symbiodinium* communities. We found consistent differences between morphs on both the macro- and micro-skeletal scale. In addition, we identified 18 candidate functional genes that relate to skeletal formation and morphology that may explain how the two morphs regulate growth to achieve their distinct growth forms. With inconclusive results in endosymbiotic algal community diversity between the two morphs, we propose that colony morphology may be linked to bleaching susceptibility. We conclude that cryptic speciation may be in the early stages within the species *P. acuta*.

## Introduction

Accurate species identification is paramount for studies addressing biological diversity, biogeography, physiology, ecology, and evolution^1^. Traditionally, taxonomy has been based almost exclusively on morphology^2–4^; however, contemporary genetic technologies have revolutionized the approach to delineate species boundaries. While morphology-based identification remains invaluable for taxonomic description and field identification, morphology-based approaches often fail to detect cryptic speciation^5, 6^. In contrast, molecular techniques are capable of resolving complex phylogenetic relationships that include hidden diversity, introgression, reticulate evolution, or hybridization^7–10^. Genetic techniques also shed light on the molecular mechanisms underlying biological processes relevant to speciation, such as local adaptation that may lead to reproductive isolation^11, 12^.

For scleractinian (reef-building) corals, species identification is extremely difficult due to high levels of intra-specific morphological variation, which was traditionally accepted as phenotypic plasticity (i.e. the same genotype can produce multiple phenotypes)^13^. This variation has the potential to blur species boundaries and confound studies due to the inclusion of multiple species rather than a single target species. The “ecomorph” concept, coined by Ernest Williams^14^ and adopted for corals by Veron and Pichon^15^, accounted for intra-specific variation by attributing it to environmental conditions that induce changes in the phenotype. In support of this concept, environmental determination of certain coral phenotypes has been confirmed through reciprocal transplant experiments^13, 16–18^. However, this terminology should be used with caution: variations in morphology may be a consequence of multiple factors, including phenotypic plasticity and genetic variation^13^. In addition, introgressive hybridization (gene flow from one species to another through backcrossing of hybrid) is common between closely related coral species^19–23^, and can blur taxonomic boundaries and lead to speciation (particularly when introgression is unidirectional). These hybridization processes, combined with cryptic speciation, can create a continuum of traits, further complicating our perceptions of species boundaries.

The common and ubiquitous reef-building coral *Pocillopora damicornis* (*sensu* Veron^24^) has been considered one of the most morphologically plastic scleractinian species. Its skeletal and physiological traits can be highly variable between different environments^13, 16, 25^. For instance, compact colonies are usually found in high wave energy sites, while more finely branching forms are common in low wave energy sites^17, 26^. Varying “ecomorphs” were therefore perceived to be members of a highly plastic, yet genetically homogeneous single species^15^. In recent years, *P. damicornis* taxonomy has been revisited, combining molecular data with micro- and macro-morphological traits to delineate species boundaries^27^. This synergistic approach to taxonomic reclassification revealed that *P. damicornis* is comprised of a group of closely related yet clearly distinct genetic lineages, representing at least eight newly defined species. The improved taxonomic scheme has helped to explain controversies over differences in life history strategies assumed for the species, such as reproductive mode or timing^27, 28^, however evidence suggests that additional cryptic species remain to be identified^27^.

On the Great Barrier Reef (GBR), *Pocillopora acuta* (described as one of the newly resolved species of the *P. damicornis* species complex^27^) occurs in high frequencies, but appears to have two distinct sympatric morphs. One morph is described by blunt, chunky branches, while the other displays a fine and spindly appearance, hereafter “chunky” and “fine.” Differential bleaching thresholds were observed between the two morphs during the mass bleaching event of 2016 on the GBR, with nearly all colonies of the fine morphotype of *P. acuta* exhibiting bleaching on impacted reefs, while those of the chunky morphotype remained largely pigmented (GT and HE, pers. obs). These two lines of evidence (differential morphology and bleaching susceptibility) suggest that *P. acuta* may be comprised of at least two cryptic species. The current study aims to assess the genomic basis of morphological traits and differential bleaching thresholds within the putative species *P. acuta*, and to evaluate any signatures of cryptic speciation or hybridization.

## Results

A total of 36 colonies of the chunky morphotype and 44 colonies of the fine morphotype were collected from three locations, separated by a maximum of 6.5 kilometers (Table 1). Both morphotypes occurred in all three locations sampled and were present in approximately equal abundance. None of the specimens of the chunky morphotype were fully bleached, and 4 out of 36 were partially bleached (11.1%). All colonies of the fine morphotype showed at least partial bleaching, with the majority (n = 35, 79.5%) fully bleached. The molecular species identification assay confirmed that 74 of the 80 colonies were *P. acuta*. Of the remaining six colonies, two failed to amplify, and four colonies identified as *P. verrucosa*.

**Table 1:** Overview of sample collection.

	Bleached	Not Bleached	Partially Bleached	Total
**Cattle Bay**				
Chunky	0	8	0	8
Fine	22	0	0	22
**Little Pioneer Bay**				
Chunky	0	16	1	17
Fine	3	0	0	3
**Pelorus Island**				
Chunky	0	8	3	11
Fine	10	0	9	19

### Sequencing Statistics and Outlier Loci

Following filtering of the raw dataset (33,026 loci), 3,179 putative SNPs were retained for the morphotype dataset, while 4,268 loci remained for the bleaching dataset. Two individuals were removed due to excess heterozygosity or missing data in both datasets. Forty-seven SNPs were detected as candidate loci under positive (divergent) selection between morphotypes, eighteen of which fell within known exon regions. Three loci were genes relevant to morphological differentiation and will be discussed below. The three relevant loci were located in exons for serine-threonine protein salt-induced kinase (SIK) 3, secreted Wnt11, and calcium ion transport and exchanger genes.

Eighteen SNP loci were detected as outliers between bleached (fine morph only) and non-bleached colonies (chunky morph only), three of which were in exons. Two of these were hypothetical proteins with no known function. The third was located in an exon in a gene for the von Willebrand factor, and is discussed below. Loci under balancing selection were not identified between morphotypes or bleaching categories.

### Population genetics

**Figure 1:**
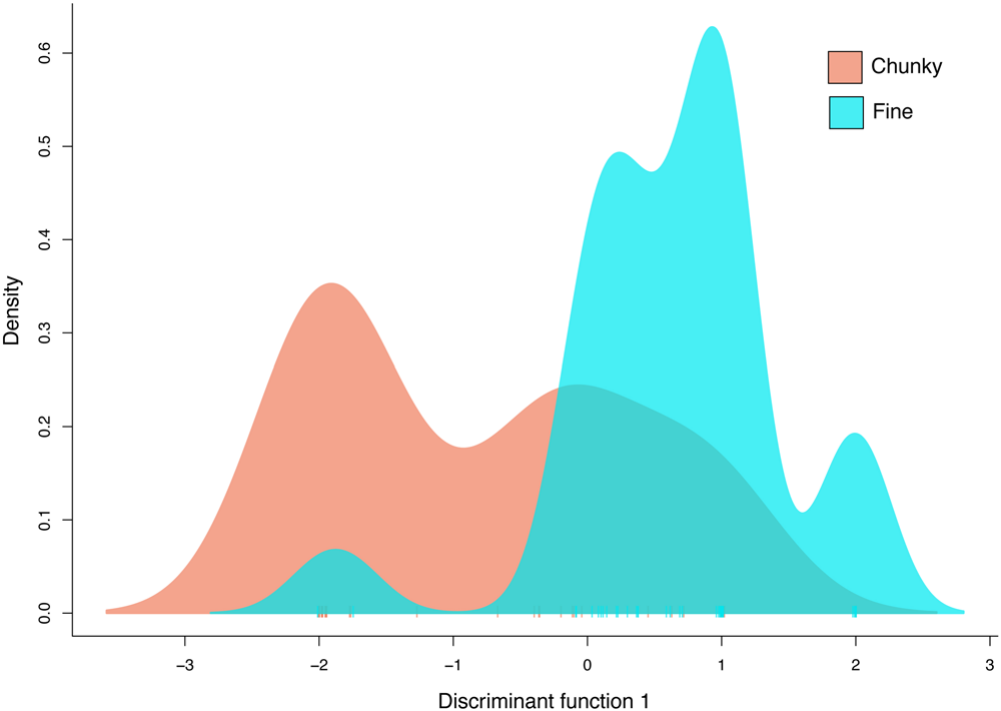
Chunky and fine morphs had distinct peaks in a DAPC analysis based on neutral loci, but still overlapped considerably.

Morphotypes were significantly different based on F_ST_ comparisons from a hierarchical AMOVA, with putatively neutral alleles nested within individual, nested within morphotype (AMOVA, overall F_ST_ = 0.075, p < 0.001). However, individuals were also significantly different within morphotypes (F_IS_ = 0.141, p = 0.001). To provide a benchmark for the level of divergence between morphotypes, in the same analysis, interspecific comparison of positive samples of *Pocillopora acuta* and its sister species *Pocillopora verrucosa* was highly different (F_ST_ = 0.712). DAPC showed strong clustering of *P. acuta* positive samples in the centre of the axis, with the chunky and fine morphs clustering on each extreme of the axis (not shown). Without *P. acuta* positive samples, the DAPC showed two strong peaks separating chunky and fine morphs, but still with great overlap (Fig. 1).

**Figure 2:**
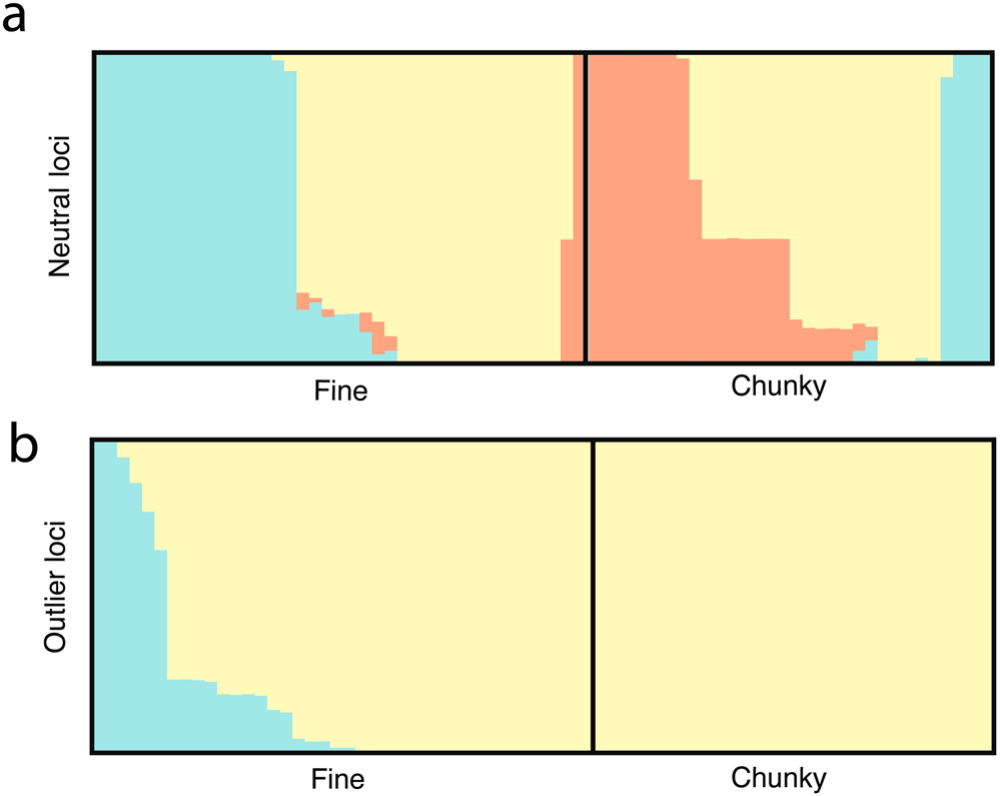
STRUCTURE bar plots depicting clustering results for chunky and fine morphotypes based on neutral loci and outlier loci, respectively. Each bar in the graphs represents an individual, with the colors representing proportional cluster membership to each of the identified clusters.

Clustering analysis followed by the Evanno method^29^ identified 3 clusters (K = 3) as the most likely number of clusters within the complete dataset (Fig. 2a). The clustering analysis showed that there is great variation among individuals within morphotypes, however the two morphs do appear to be distinctly different overall (‘green’ cluster is more common in fine; ‘red’ cluster is more common in chunky, Fig. 2a). This pattern mirrors the F-statistics: the morphotypes contain markedly different elements but individuals vary greatly within types. When considering the outlier loci alone, two clusters (K = 2) were identified as the most likely number of clusters in the dataset (Fig. 2b).

### Symbiodinium

A total of twelve ITS2 clades were detected from 51 samples. All ITS2 fingerprint types were within clade C. Each morph contained one exclusive, numerically rare subclade: subclade C4 was only found in the chunky morph, representing 0.2% of community diversity. Alternately, subclade Cspd was only found in the fine morph, representing 0.1% of community diversity. Similar patterns were found across bleached and non-bleached communities (Supplementary Fig. S1).

**Figure 3:**
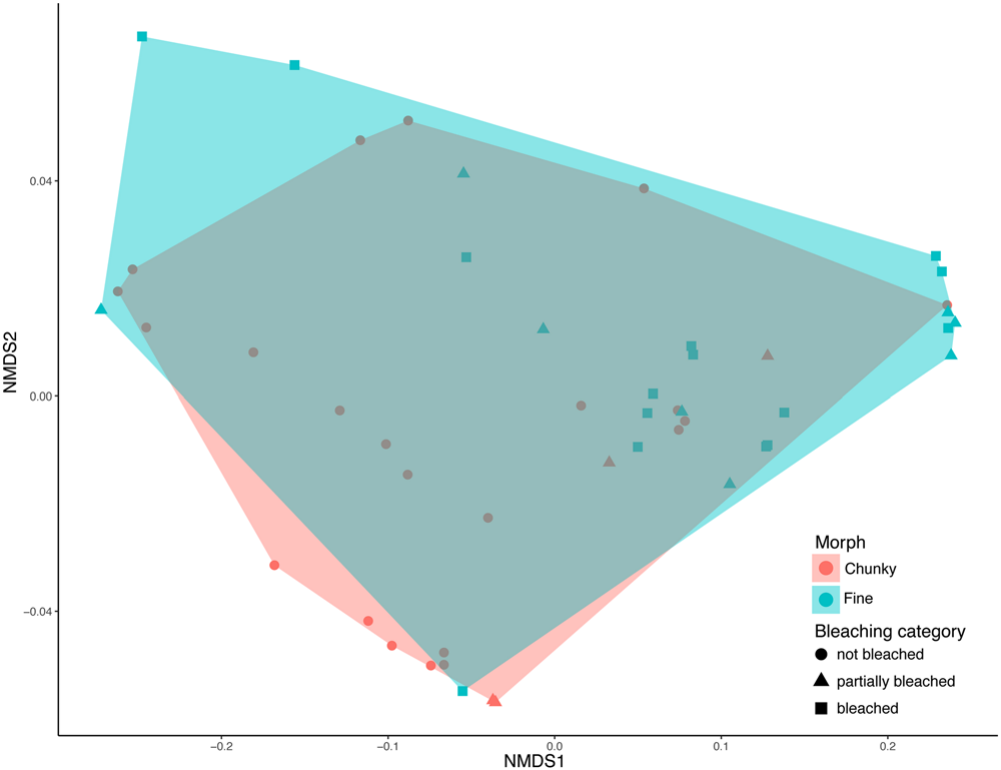
Clustering of multivariate *Symbiodinium* community composition data clouds in each coral sample visualized using non-parametric multidimensional scaling (nMDS).

Shannon diversity index did not differ significantly between morphotypes (ANOVA, F = 1.5879, p = 0.2138) or bleaching status (ANOVA, F = 1.8223, p = 0.1731) (n = 49). There was no significant difference in beta-dispersion for morph or bleaching (ANOVA, F = 0.4507, p = 0.5053; F = 0.3504, p = 0.7062 respectively) and thus PERMANOVA results should be robust to calculate differences in the centroids of the multivariate data clouds. While diversity, evenness, and richness did not significantly differ between morphs or bleaching categories, PERMANOVA indicated a significant difference in global community structure between both morphs and bleaching categories. Morphotype was found to be a significant factor to explain community composition (PERMANOVA, R^2^ = 0.1069, p = 0.007), as was bleaching category (PERMANOVA, R^2^ = 0.1129, p = 0.022), despite no obvious separation of the data cloud clusters by morphotype or bleaching category (Fig. 3).

### Morphology

**Figure 4:**
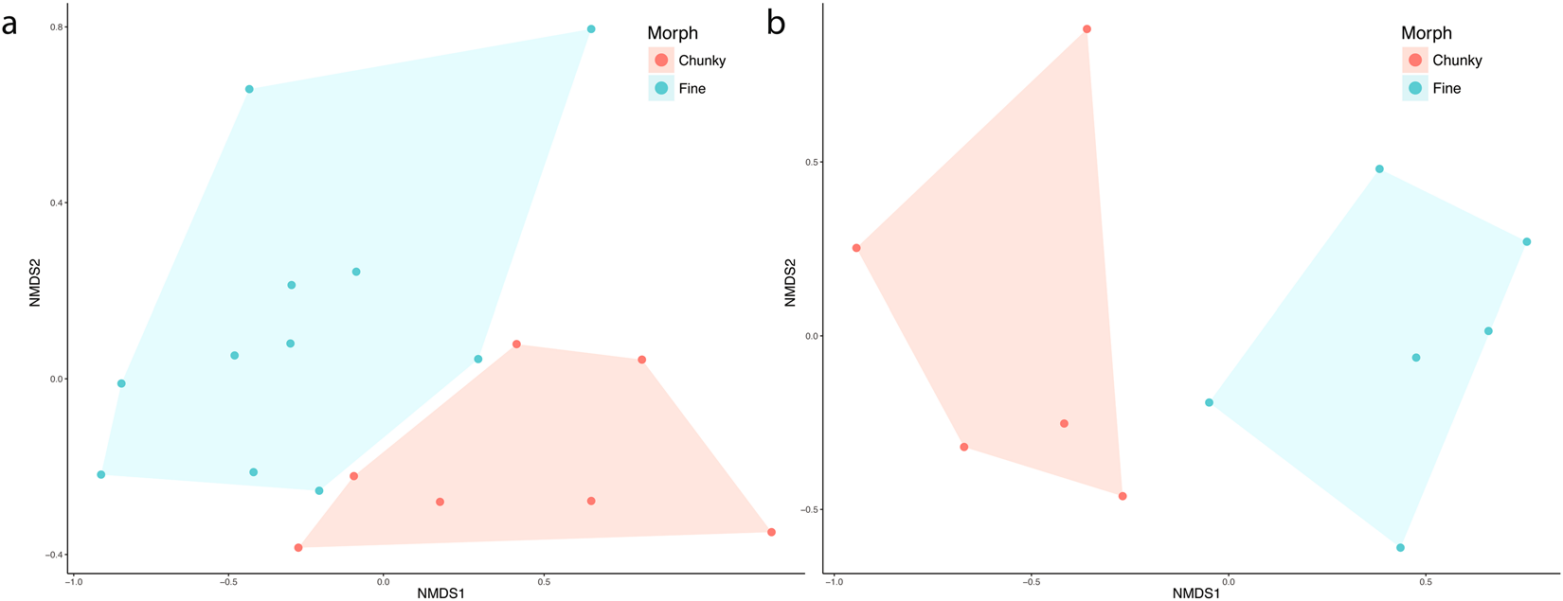
(**a**) nMDS plot of microskeletal features. Points represent the multivariate position of each corallite measured. (**b**) nMDS plot of macroskeletal features. Points represent branches measured.

Differences between morphs in micro and macro-skeletal features were significantly explained by morphotype (micro: PERMANOVA, R^2^ = 0.308, p = 0.007; macro: PERMANOVA, R^2^ = 0.326, p = 0.005). However, there was also significant variation in micro-skeletal features among colonies within morphotypes (PERMANOVA, R^2^ = 0.179, p = 0.024). Nonparametric multidimensional scaling of both micro- and macro-skeletal features showed distinct clustering by morphotype (Fig. 4), with no overlap in the multivariate data clouds.

**Figure 5:**
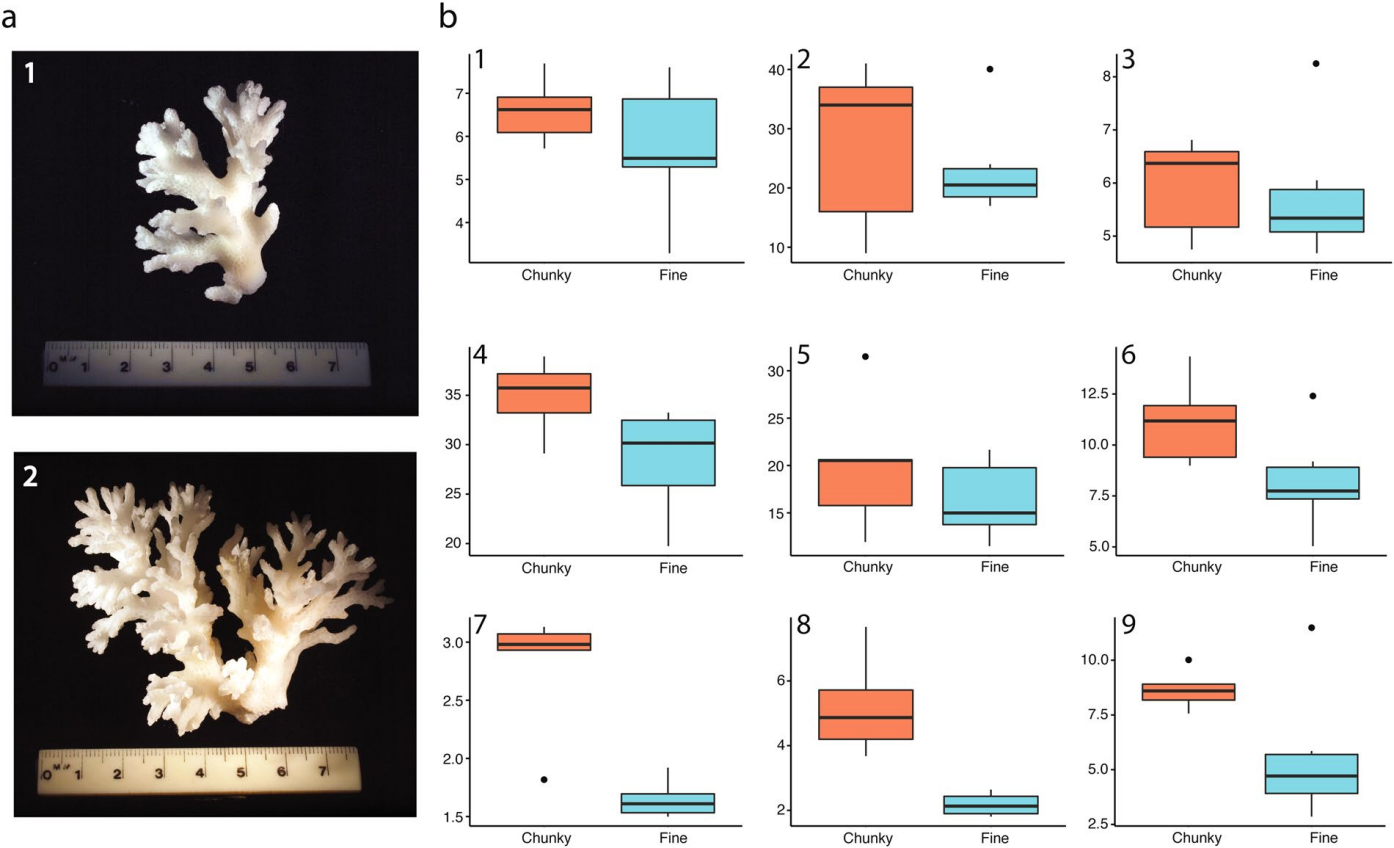
(**a**) Photos of “type” specimens for each morphotype; photo 1 is chunky morphotype, photo 2 is fine morphotype. (**b**) Box plots of nine macroskeletal variables. Colors indicate morphotypes (orange: chunky; blue: fine); all Y-axes are in millimetres. Plots B1-B9 represent morphometric variables^27^ (Supplementary Table S1).

**Figure 6:**
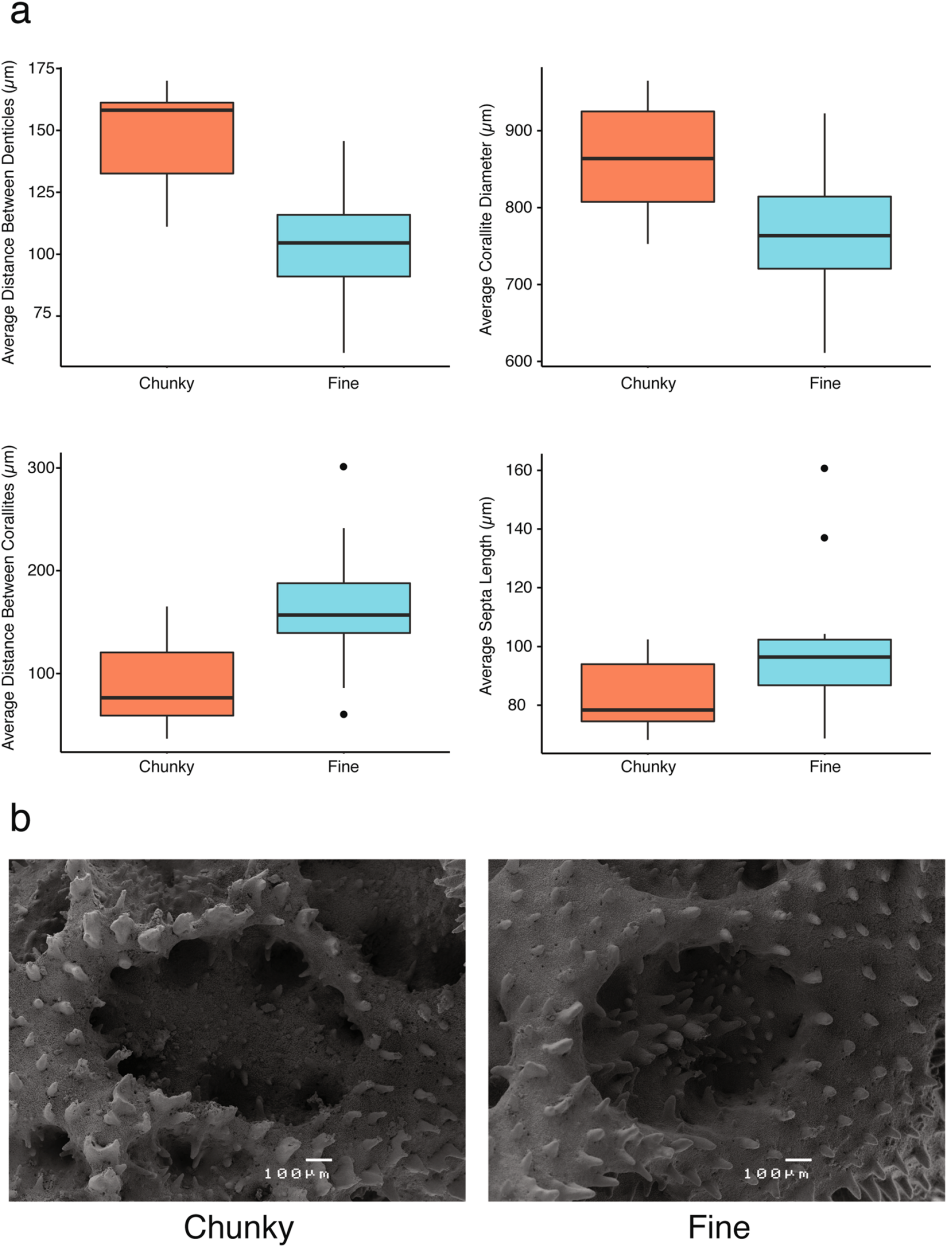
(**a**) Box plots of four microskeletal variables. Colors indicate morphotypes (orange: chunky; blue: fine). (**b**) Scanning electron microscope photos of characteristic corallites for each morphotype.

Overall, macroskeletal features of the chunky morph were significantly larger and more spaced out than the fine morph (Fig. 5). Microskeletal features of the chunky morphotype showed a trend of greater corallite diameter and longer distance between denticles than the fine morphotype. In contrast, the fine morphotype generally had more spaced out corallites (i.e. cup-like skeleton of each polyp) and longer septae (i.e. dividing skeletal wall) than the chunky morphotype (Fig. 6).

## Discussion

The results of our study show that genetic divergence has occurred between two sympatric morphotypes of a single putative species with differential bleaching susceptibilities, providing evidence of an unresolved taxonomic structure. The genetic and morphological traits observed in the present study do not align with established species boundaries^30–32^, highlighting that corals do not necessarily adhere to traditional taxonomic and systematic patterns^33^. It is possible, therefore, that potential incorrect identification within *Pocillopora acuta* in past studies has led to false conclusions regarding biological patterns and processes through the unintentional inclusion of multiple species in assumed single-species studies. Incorrect identification can also confound the ability to combine knowledge derived from multiple sources. This study provides important evidence that further taxonomic resolution is required in order to avoid such incorrect conclusions.

Despite a lack of clear genetic partition between the two morphotypes of *P. acuta* examined in this study, there is evidence for morphological differentiation within the species, as indicated by significantly different micro- and macro-skeletal structures. While more intensive skeletal sampling is needed to confirm that morphological traits quantitatively align with the patterns detected in our type specimens, as few as four colonies have been used in past studies to delineate a novel taxon, *Pocillopora bairdi* ^27^. Despite the need for more sampling, the two morphs are distinct enough that visual inspection of photographs provides strong evidence for differentiation.

In addition to morphological differentiation, significant differences between pairwise F_ST_ suggests that morphs are distinct. However, high intra-morphotype genomic variation is present within both morphotypes, preventing a clear split between the two. Morphological modifications driven by divergent natural selection may be accompanied by only low levels of genetic differentiation^1^. Low (albeit significant) levels of differentiation between the two morphs examined here suggest that if speciation is occurring between these morphs, it may be recent and ongoing, and thus they may not have diverged considerably due to the lack of an absolute reproductive isolation. Corals are known to hybridize with congeneric or even confamiliar species^19–23^. Consequently, sympatric speciation may be a slow evolutionary process in scleractinians, with introgressive hybridization potentially decelerating genetic divergence. The three genetic clusters detected in our structure analysis, combined with overlapping DAPC plots suggest that there has been recent gene flow between the morphotypes, and the three clusters may represent three cryptic genetic lineages: two pure-bred (i.e. chunky and fine) and an intermediate hybrid population. Further investigation with more robust outgroups is required to determine if these lineages represent species, sub-species, or population level divisions.

While the population genetic analyses based on outlier loci did not provide clear discrimination between the morphotypes, the lack of differentiation may be attributed to continued contact between the morphotypes, thus preventing differentiated loci to completely diverge. Nevertheless, genetic differentiation between the two morphotypes of *P. acuta* was driven in part by a small number of functional outlier loci on exons, indicative of divergent adaptive strategies in the same habitat. Lositan was determined to be the program best fit to our study system, but it is worth noting that implementation of other outlier detection methods (such as BayeScan, OutFLANK, or Arlequin) may yield different results and merit further exploration. Using Lositan, outlier SNPs were detected on genes linked to morphological development. For example, one gene that showed signals of selective differentiation between morphs was the Wnt11 gene, a gene involved in the major signaling pathway called wingless (Wnt). This pathway was first discovered in 1982 as an oncogene in mice, and nearly simultaneously discovered to specify wing development in *Drosophila* (history of the pathway is reviewed by Nusse and Varmus^34^). The Wnt pathway is involved in pattern specification, cell fate determination, axis determination, tissue regeneration, and biomineralization within a suite of organisms such as *Drosophila* and the frog genus *Xenopus* ^35–37^. In Scleractinian corals, Wnt genes play a role in early development and are involved in oral/aboral axis determination (especially in axial patterning of larvae), tentacle formation, and body patterning^38, 39^. Small changes in Wnt genes have been suggested to play a key role in cnidarian speciation: perturbations to the Wnt pathway result in dramatic downstream effects on cnidarian axial patterning and body plan, alterations which form a basis upon which natural selection may act^40^. In vertebrates, Wnt genes are involved in the regulation of biomineralization and osteogenesis^41^, and it is possible that the Wnt signaling pathway may play a role in skeleton formation in calcifying corals. Wnt genes seem to be up-regulated in branch tips, and the Wnt pathway is suggested to be the key signaling pathway regulating differential branch patterning between *Acropora* species^37^. It is possible that genetic differentiation in the Wnt gene between morphotypes has led to mutations in body patterning and may explain the differences in branch tip shapes found in the current study. Interestingly, genes involved in stress response may interact with Wnt genes^42^ and this interplay could explain the link between morphology and bleaching, however more quantitative techniques such as gene expression analysis or digital PCR are needed to investigate this hypothesis.

Another locus that drives divergence between the two *P. acuta* morphs was serine-threonine protein salt-induced kinase (SIK) 3, a gene involved in the morphogenesis of anatomical structures. While the function of the gene has not been identified for corals, serine-threonine kinases have been shown to regulate morphogenesis and development in a variety of organisms from bacteria and yeasts to *Drosophila* ^43–46^. In *Drosophila*, the main pathway which specifies growth and size control is regulated by SIK3 kinases^47^. It is possible that this gene may similarly regulate aspects of morphogenesis and development in corals, such as branch size determination.

A calcium ion transport and exchanger gene also showed significant differentiation between morphotypes. While several genes are involved in calcification^48^, exchanger genes are involved in mediating the movement of calcium ions, a process vital to calcification and skeletal formation. Selection upon a single gene may indicate that a mutation occurred, and may cause downstream changes in morphology, such as skeleton thickness. While the direction of selection cannot be determined, the combined evidence from observed bleaching patterns suggests that the chunky morph is better adapted to thermal stress, and perhaps positive selection is occurring upon this gene. Alternatively, the fine branching form may optimize resource allocation to skeletal growth, which may inadvertently be linked to increased bleaching susceptibility in this molecular pathway.

Between bleached and non-bleached colonies, the only outlier SNP falling in a known exon was a von Willebrand factor domain protein, an adhesion glycoprotein that plays a role in the structure and adhesion of cells. Von Willebrand factor proteins are well studied in humans, but in corals, the role of these proteins is not fully understood. There is some evidence that the gene is involved in skeletal organic matrix formation and cell adhesion in corals, and appears to be upregulated in both pre-settlement larvae and in immune-challenged adult colonies as part of the allogenic response^49–51^. This response is known to play an important role in the establishment and maintenance of symbiosis^52^. The strong divergent selection on this gene detected in our study indicates that it may contribute to thermal resilience.

In addition to genetic signals of the coral host, bleaching susceptibility of scleractinian corals is largely influenced by the composition of the endosymbiotic algal community^53, 54^. In our study, the contradictory results of the ANOVA and PERMANOVA are inconclusive of whether symbiont communities vary between the two morphs, and therefore cannot be reliably linked to bleaching in this study. The non-concordance of test results may be due to differences between the two statistical methods: PERMANOVA is sensitive to all pairwise differences in *Symbiodinium* clades, while ANOVA evaluates only the difference in overall diversity as estimated by the Shannon Index.

In the current study, we were also unable to evaluate differences between original (i.e. pre-bleaching) versus remnant communities. Thermally sensitive clades are likely to be the first rejected from the coral host tissue, and may constitute a greater proportion of the original community. The similarity between *Symbiodinium* community proportions in this study may be attributed to a vast difference in absolute value of symbiotic cells. To clarify the role of *Symbiodinium* communities in bleaching susceptibility of *P. acuta* morphotypes, future studies should repeatedly sample tagged colonies before and over the course of a bleaching event. Potential intragenomic variation may also be interrogated using bioinformatics methods^55^.

While the outlier SNP for von Willebrand factor may contribute to bleaching susceptibility, it is unlikely that a single gene would control a colony’s response to thermal stress. Indeed, Bay and Palumbi^56^ found that a suite of at least 100 genes were correlated with coral thermal resistance in colonies occurring in a thermally highly variable versus a moderately variable habitat. Broader sequencing with more restriction enzyme combinations may more fully capture SNPs associated with bleaching patterns not detected in the current study. Gene expression studies, such as weighted gene co-expression network analysis (WGCNA), may also identify candidate genes that may be related to thermal tolerance.

In the absence of obvious bleaching-linked functional SNP differences in the host genome, and inconclusive results on the role of symbiont diversity in bleaching tolerance between morphs, we propose that morphological variation, driven by functional outlier loci, may be a link to explain differential bleaching patterns observed in this study. It has been theorized that a fine branching structure optimally disperses and reflects light^57^. Colonies with long, thin branches are generally found in deep habitats, as this skeletal formation allows access to higher mass-transfer and irradiance^58^. However, in a shallow habitat (such as the habitat where our morphs are found), this branching pattern may lead to supra-optimal light transfer, increasing irradiance stress and potentially leading to bleaching in summer heatwaves^57, 59^.

A fine branching growth form is also a fragile one – if indeed the two morphs examined in this study were “ecomorphs,” we would not expect to find the fine form in shallow habitats, as high wave energy discourages this colony growth form^60^. Our discovery of the fine morph in shallow habitats lends further support that *P. acuta* morphotypes are genetically predetermined, and are not a result of phenotypic plasticity. In addition, the fine growth form may be typical of deeper habitats, but in the absence of major regional thermal stress events over the past two decades (since 1998), colonies of fine growth form may have opportunistically settled on the upper reef slope. Future work could evaluate if the fine growth form continues to be found in high energy habitats after the major bleaching event of 2016.

In conclusion, we have established a functional genomic basis for consistent morphological differentiation between two distinct sympatric morphotypes of a single putative species. In addition, we have established a link between morphology and bleaching susceptibility regardless of endosymbiont community composition. Consistent patterns in morphology and corresponding genetics within *P. acuta* warrant further investigation to determine whether cryptic speciation is in its early stages, or we are witnessing introgressive hybridization.

## Methods

### Sample collection

**Figure 7:**
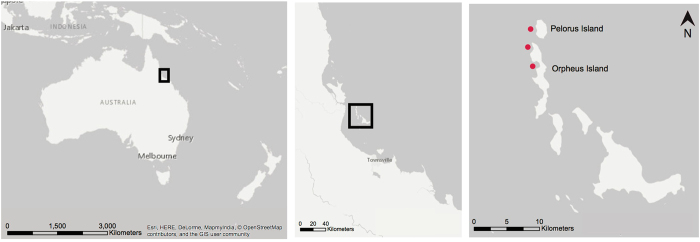
Map of collection locations. Map generated in ESRI ArcMap version 10.4.1 (http://desktop.arcgis.com/en/arcmap/).

Coral nubbins were sampled from 80 colonies across three locations in the Palm Island Group, central GBR in March 2016 at a depth of 2–5 m (relative to chart datum) from fringing reefs at Cattle Bay (n = 30) and Little Pioneer Bay (n = 20) (Orpheus Island); and a leeward site off Pelorus Island (n = 30) (Fig. 7). Only colonies occurring in similar habitats, depths and flow regimes were collected to ensure morphology was not variable due to plasticity alone. Colonies were identified in the field as *P. acuta* based on gross morphological descriptions^27^. Samples of both morphotypes were collected haphazardly and fixed in absolute ethanol, while ensuring all present bleaching categories (fully bleached, partially bleached, not bleached) were collected. In addition to nubbin samples, two full colonies representative of each morphotype were collected for skeletal analysis.

### Species Identification

Total genomic DNA (gDNA) was extracted following an adaptation of a published protocol^61^ (Supplementary Methods). Species identification was ascertained following the methodology of Torda and colleagues^62^. Positive control samples of *P. acuta, P. damicornis, and P. verrucosa*, collected from the same sites four years prior to the current study^62^, were used for comparison and outgroups. Samples collected in the current study that did not identify as *P. acuta* (n = 4) were combined with positive control outgroups for further SNP analysis.

### SNPs library preparation and data filtering

Following species identification, gDNA extracts were sent to Diversity Arrays Technology (DArT, Canberra) for genome-wide restriction-site associated DNA (RAD) sequencing and identification of single nucleotide polymorphism (SNPs) loci. The DArT proprietary method^63^ is similar to the widely-applied RADseq methodology^64^, and combines complexity reduction methods and the Illumina HiSeq 2500 next generation sequencing platform. The PstI-HpaII complexity reduction method was used, with fragments amplified by polymerase chain reaction (PCR; Supplementary Methods). The single read sequencing was run for 77 cycles. The sequences for each lane were filtered to exclude poor-quality sequences, applying more stringent criteria to the barcode region to ensure reliable sample identification. Approximately 2,500,000 (+/− 7%) sequences per sample were used for marker calling. Identical sequences were collapsed and used in a secondary proprietary analytical pipeline for single nucleotide polymorphism (SNP) calling.

Raw, scored SNP data was filtered to exclude loci with a call rate <0.95 and an E-value of 999 (accuracy of match to a reference pocilloporid genome; Vidal-Dupiol *et al*., in review). In two separate pipelines, samples were grouped by morphotype or bleaching category, and SNPs were quality controlled as follows. Monomorphic loci, as well as loci with a minor allele frequency (MAF) of 2% or less were excluded, as genotyping error is thought to be highly likely below this threshold. One of each pair of SNPs that occurred within 50 base pairs of each other was retained, keeping the locus with a higher MAF. Loci that showed significant deviation from Hardy-Weinberg Equilibrium (p < 0.001) across all populations, and loci exhibiting complete linkage disequilibrium as calculated in PLINK v1.072 were excluded. Finally, individuals with excess heterozygosity (>0.1) and missing data (>0.3) were removed. The pruned datasets were used for all downstream analyses comparing morphology or bleaching category with SNPs genotype. When bleaching categories were compared, only fully bleached and fully pigmented colonies were included to better distinguish the underlying factors that may be contributing to differential bleaching susceptibilities.

### Outlier loci detection

Outlier loci (loci under potential balancing or divergent selection) between the two morphotypes and between the bleaching categories were detected in Lositan Selection Workbench^65^. Lositan employs a coalescent-based simulation approach to identify loci exhibiting unusually high or low F_ST_ values, whereby observed F_ST_ values are compared to values expected under a neutral evolutionary model. It has been demonstrated that Lositan performs better than other detection methods, including BAYESCAN, when signals of selection may be weak, such as in populations experiencing ongoing contact or hybridisation^66^. Initial runs of 50,000 simulations were performed on the pruned datasets described above, using the calculated mean F_ST_. Because F_ST_ can be skewed by the inclusion of outliers, the loci identified as outliers (outside the 99% confidence interval) in the first run were then excluded from a second run in order to gain a more refined estimate of the true neutral F_ST_. This refined neutral F_ST_ estimation was used as the basis for 100 runs of 50,000 simulations each over the complete datasets. To prevent the inclusion of false positives, only the loci falling outside the 99% confidence interval in all 100 runs were concluded to be candidate outliers.

The detected outlier SNPs for both the morphological and bleaching datasets were mapped to an annotated *Pocillopora* reference genome (Vidal-Dupiol *et al*., in review), and functional annotation was explored. Biological function was evaluated based on the UniProt annotation database and a review of literature.

### Population genetic analysis

For population-based genetic analysis, only neutral loci were included in all analyses, and we considered morphotypes as putative “populations.” Hierarchical organization of genetic variation was assessed using an analysis of molecular variance (AMOVA) based on F_ST_ value comparisons within and among morphotypes as implemented in GenoDive v2.0b27^67^. In a single analysis, alleles were nested within individual, nested within morphotype. Positive samples of *P. verrucosa* were included in the analysis to provide a benchmark for the level of interspecific differentiation. The analysis was based on an Infinite Allele Model with 9999 permutations. F-statistics were obtained through bootstrapping over all loci. Discriminant analysis of principal components (DAPC^68^) was performed to visualize clusters using a priori defined groups (i.e. morphotypes). The number of principal components retained was optimized for a-score using the package adegenet version 1.3-1^68–70^ in R Studio version 0.99.903^71^. The analysis was performed in two iterations: one with outgroups and one without, in order to contextualize and improve cluster membership resolution of the target samples.

Bayesian clustering analysis was implemented in STRUCTURE v2.3.2^72^ using all loci to identify the number of genetic clusters and to assign individuals to clusters (K = 1–8) following the methodology of Ladner and Palumbi^22^. Iterations were aligned in CLUMPP^73^ and visualized in DISTRUCT^74^. STRUCTURE and k-selection was repeated using outlier loci only, as analysis of these loci lend more power to detect recent divergences between populations^75–77^.

### Symbiodinium ITS2 Analysis

To determine if *Symbiodinium* community composition was a driver of bleaching patterns observed, the same DNA extracts described above were used to amplify the internal transcribed spacer region 2 (ITS2) of *Symbiodinium* ribosomal DNA. PCRs were run in triplicates for each sample, using 5 μl KAPA HiFi Hot Start Ready Mix, 2μl each of 1 mM primer, and approximately 50ng/1μl DNA template for a total volume of 10μl. The PCR conditions in a Kyratec Thermocycler were as follows: initial 3-minute denaturation at 95 °C followed by 25 cycles of 30 s at 95 °C (denaturation), 30 s at 55 °C (annealing), and 30 s at 72 °C (extension), finished with 5 minutes at 72 °C for final extension and held at 4 °C. For each sample, triplicate PCR products were pooled and separated on a 1% TBE agarose gel in 0.5% TBE buffer to visualize successful amplification. Nineteen of 76 samples did not successfully amplify and were excluded from further processing and analysis. PCR products were sent to the Ramaciotti Centre for Genomics (UNSW, Australia) for sequencing using Illumina Mi-Seq paired-end technology, with 2 × 300 bp read length. A total of 2,243,453 reads with a mean of 38,024.6 reads per sample (n = 57) were provided as demultiplexed FASTQ files, which were processed through a custom pipeline (Supplementary Methods).

Statistical analysis was performed in R Studio^71^ with the vegan^78^ and nlme^79^ packages. A Bray-Curtis dissimilarity matrix was created and clustering was visualised using non-parametric multidimensional scaling (nMDS). Shannon Diversity index, species richness, and species evenness were calculated and compared between morphotypes and bleaching categories using one-way ANOVA. A non-parametric permutational multivariate analysis of variance (PERMANOVA) was used to test differences in ITS2 communities^80, 81^. Homogeneity of multivariate dispersion was first tested to ensure data fit the assumptions of the model^80^, and two iterations of PERMANOVA were carried out with 9999 permutations, with community diversity as the dependent variable, and morphotype or bleaching category as the explanatory variable, respectively.

### Microskeletal morphology

Two colonies of each morphotype were selected as type specimens and were bleached in 50–100% sodium hypochlorite. Small fragments of approximately 5mm were cut using a motorized high-speed cutting disc (Dremel). The fragments were cleaned with a compressed air-duster and mounted on aluminum stubs before vacuum gold coating. The samples were viewed in a JEOL JSM5410LV scanning electron microscope (SEM) at The Advanced Analytical Centre (James Cook University, Townsville). Resulting images were edited for contrast and light correction in the program SemAfore v5.2 (http://semafore.software.informer.com), and micro-skeletal features were measured in Adobe Photoshop. The images were measured for the following corallite characters (chunky morph n = 7 corallites; fine morph n = 11 corallites): (i) the average of the longest and shortest diameter across a single corallite; (ii) the average distance between a single corallite and its three closest neighbors; (iii) the average distance between three sets of haphazardly chosen denticles on the coenosteum; and (iv) the length of the longest septa projecting inwards; as recommended by Maté^82^, Torres^83^, and Marti-Puig and colleagues^32^.

To compare micro-morphology between the two putative morphotypes, a PERMANOVA was carried out with log-transformed variables and Euclidean distance in the vegan package in R Studio with morphotype as a fixed variable, colony as a random variable, and corallite as the unit of replication. The clustering of the two morphotypes was visualized using nMDS.

### Macroskeletal morphology

Nine macro-skeletal features were measured for 3 haphazardly selected branches from each of two colonies per morphotype using a digital Vernier caliper. Variables measured were as per Schmidt-Roach *et al*.^27^ (Supplementary Table S1). After confirming homogeneity of multivariate dispersion, non-parametric PERMANOVA was performed in R Studio using the vegan package^78^. The two-factor nested model implemented log-transformed variables and Euclidean distance, morphotype as a fixed variable, colony as a random variable, and branch as the unit of replication. An nMDS was carried out to visualize clusters of the two morphological groups.

## Electronic supplementary material

Supplementary Material
