# Supplementary material for: The molecular basis of differential morphology and bleaching thresholds in two morphs of the coral Pocillopora acuta
Source: Sci Rep. 2017 Aug 30;7:10066. doi: 10.1038/s41598-017-10560-2 (PMC5577224; doi:10.1038/s41598-017-10560-2)
Supplement: Supplementary file 1 — Supplementary Material [file 41598_2017_10560_MOESM1_ESM.pdf]

**The molecular basis of differential morphology and bleaching thresholds in two morphs of the coral *Pocillopora acuta***

**Supplementary Material**

**Hillary Smith*[1,2,3,4], Hannah Epstein[1,2,3,4], and Gergely Torda[1,2]**

[1]ARC Centre of Excellence for Coral Reef Studies, James Cook University, Townsville, Queensland 4811, Australia
[2]Australian Institute of Marine Science, PMB 3, Townsville, Queensland 4810, Australia
[3]College of Science and Engineering, James Cook University, Townsville, Qld, Australia
[4]AIMS@JCU, Australian Institute of Marine Science and James Cook University, Townsville Qld 4811

*Corresponding author: Hillary Smith – hillary.smith@my.jcu.edu.au

**Supplementary Figure S1.**

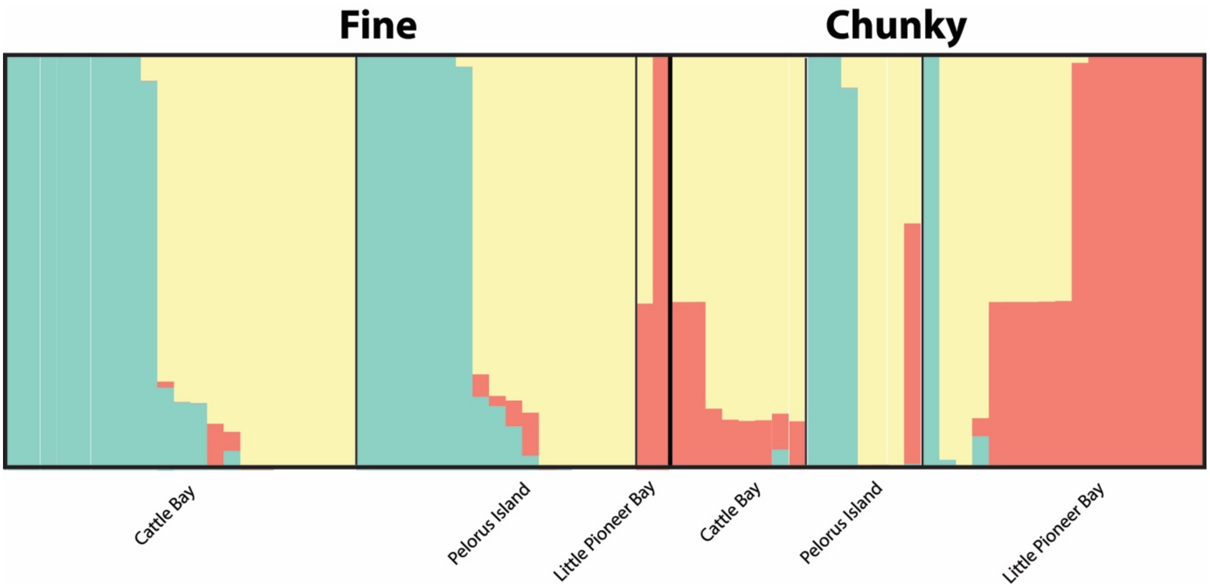


Figure 1. Proportional cluster membership sorted by population within morphotype, based on neutral loci alone.

**Supplementary Figure S2.**

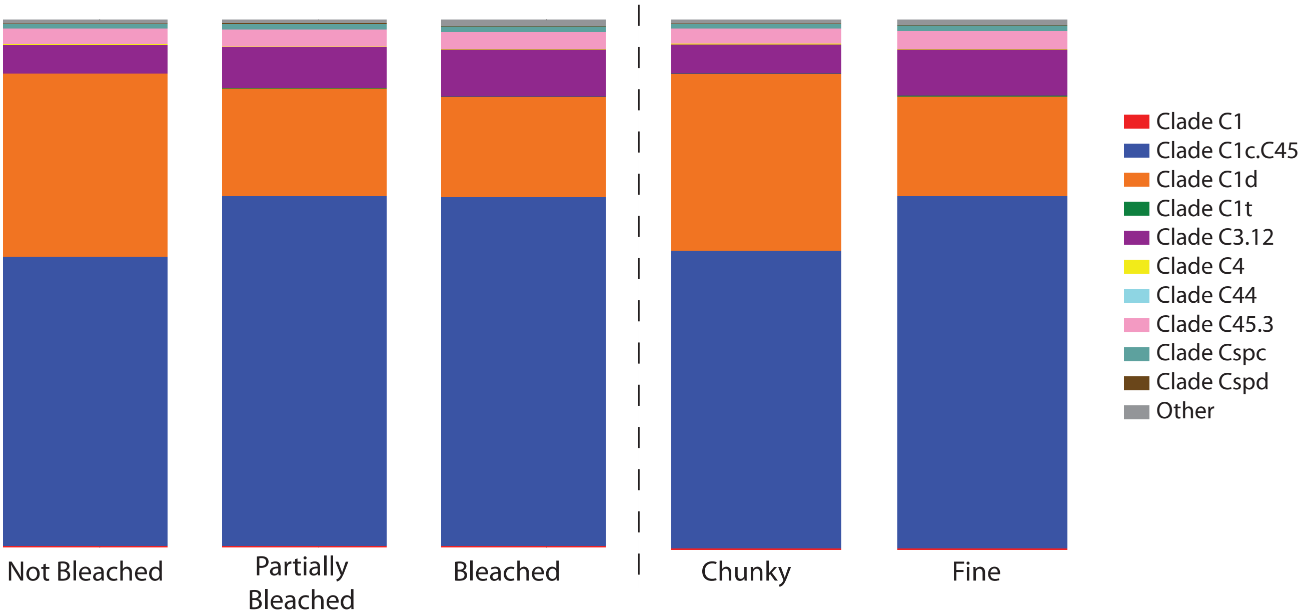


Figure 2. Proportional composition of *Symbiodinium* communities in *Pocillopora acuta* colonies within bleaching categories and morphotypes.

**Supplementary Table S1.** Macroskeletal morphological measurements analysed, as per Schmidt-Roach et al. (2014).

| | |
|---|---|
| **1** | The maximal diameter of the most distal branchlet 1 mm under tip, at most distal branch |
| **2** | The minimal diameter of most distal branchlet 1 mm under tip, at most distal branch |
| **3** | Distance between most distal branch tip and the base of the most distal ramification of a main branch with secondary branching |
| **4** | Distance between most distal branch tip and the base of the second most distal ramification of a main branch with secondary branching |
| **5** | Maximal diameter half way between most distal branch tip and the base of the most distal ramification of a main branch with secondary branching |
| **6** | Maximal diameter of branch at this most distal ramification of a main branch with secondary branching |
| **7** | Minimal diameter of branch at this most distal ramification of a main branch with secondary branching |
| **8** | Number of primary branches (branchlets/verrucae) between tip of most distal branch and most distal ramification of a main branch with secondary branching |
| **9** | Length of longest primary branch/branchlet/verrucae between tip of most distal branch and most distal ramification of a main branch with secondary branching |

**Supplementary Method: DNA extraction**

DNA extraction method.

<u>Extraction buffer:</u>

100 mM Tris pH 9.0
100mM EDTA
1% SDS
100 mM NaCl
Milli Q water
10 mg/mL Proteinase K

1. Place coral nubbin in 1.5mL microfuge tube.
2. Add 0.25 mL extraction buffer to each tube and vortex.
3. Place samples in 65°C water bath to incubate for 1-2 hours.
4. Add 62.5 uL of 5M KOAc to tube for a final concentration of 1M.
5. Incubate on ice for 30 minutes.
6. Centrifuge for 15 minutes at top speed.
7. Transfer clear supernatant to 1.5mL Eppendorf tube. If floating material remains, re-centrifuge. Note the amount of supernatant transferred.
8. Add 0.8x volume isopropanol to precipitate. Invert gently and let stand 15 minutes at room temperature.
9. Centrifuge for 15 minutes at top speed.

10. Carefully tip off supernatant.
11. Wash the pellet with 50ul 70% ethanol and re-spin briefly.
12. Carefully remove the supernatant.
13. Air dry. Ethanol should evaporate, as it prevents DNA from eluting. However, it must not be over-dried, as this will prevent DNA from eluting as well.
14. Resuspend pellet overnight in 15ul Tris 10mM pH 7.5, EDTA 1 mM (or Milli Q water).

**Supplementary Methods: PCR amplification for DArT Sequencing**

- 94° C for 1 min.
- 30 cycles of: 94° C for 20 sec
  58° C for 30 sec
  72° C for 45 sec
- 72° C for 7 min

## Supplementary Methods: ITS2 analysis

***Symbiodinium ITS2 Pipeline:***

Read quality was visualized using FASTQC[1]. Samples which had fewer than 100 total sequences or a drop in quality score below 28 within the first 100bp in either the forward or reverse read were discarded (n=8), as these criteria are indicative of low quality sequencing. Raw sequences were trimmed using conservative criteria in Trimmomatic[2], and all sequences with fewer than 170 bases were discarded. The program PEAR version 0.9.8[3] was used to merge paired-end reads, and putative chimeric sequences were removed using the USearch61 method[4] as implemented in QIIME[5]. Operational taxonomic units (OTUs) were chosen implementing the uclust method[4] in QIIME[5] specifying a minimum of 99% similarity to the reference database developed by Arif and colleagues[6], then dereplicated. From the resulting OTU table, sequences comprising less than 5% of all sequences were removed from further analysis.

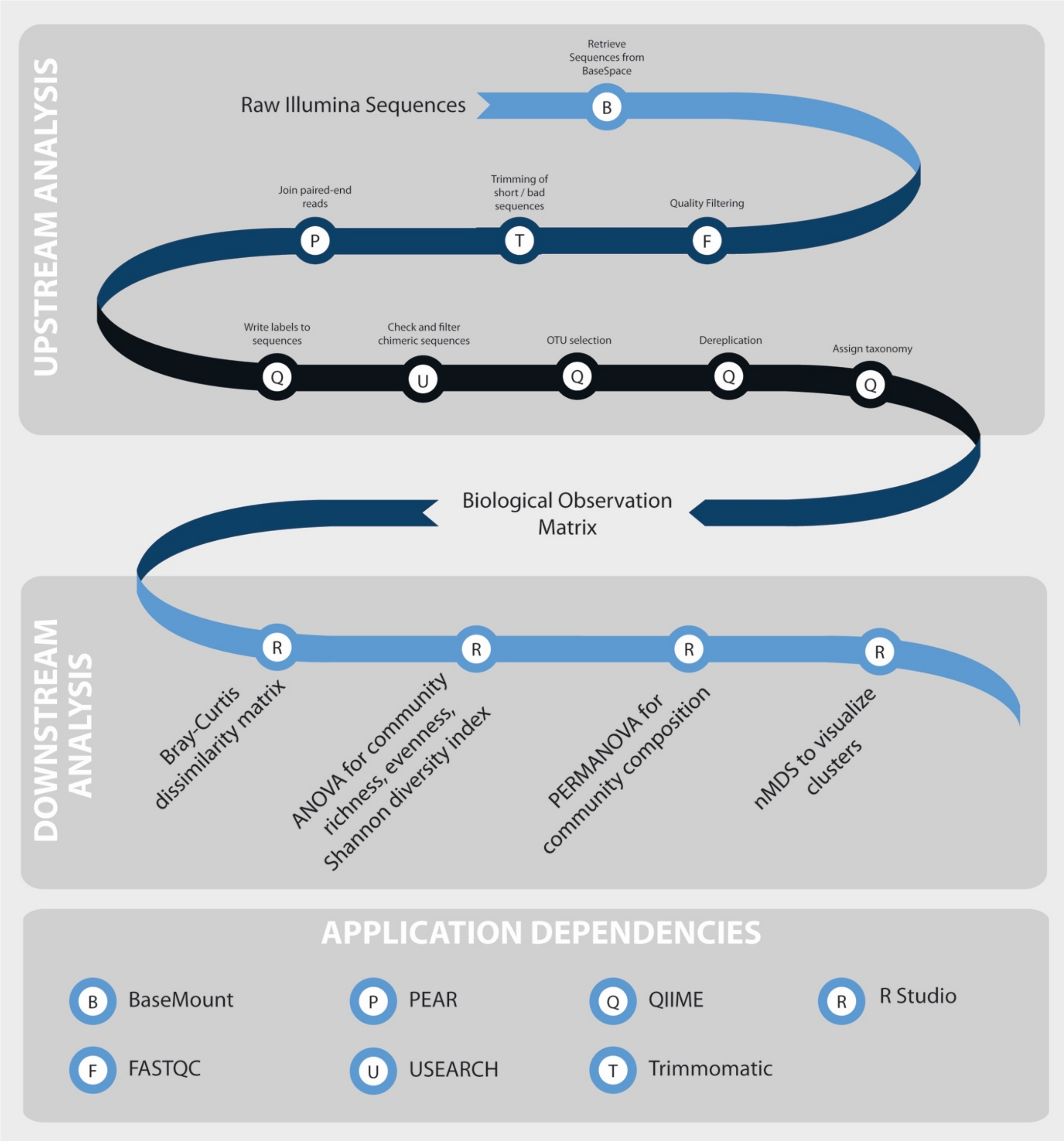


***Script for upstream analysis***

```
#Trimmomatic
# -trim 5 and 3' ends until q >= 3
# -trim from 3' end until [qqqq] > 15
# -discard sequences < 170 bases

java -jar /export/home/f-k/hsmith/HSITS/Trimmomatic-0-
2.36/trimmomatic-0.36.jar \
PE -threads 16 -phred33 \
-trimlog /export/home/f-
k/hsmith/HSITS/1_Sequences/1_adapterclipped/${i}_trimlog.txt \
/export/home/f-
k/hsmith/HSITS/1_Sequences/00_raw/*_S${i}_L001_R1_001.fastq.gz
\
```

```
/export/home/f-
k/hsmith/HSITS/1_Sequences/00_raw/*_S${i}_L001_R2_001.fastq.gz
\
/export/home/f-
k/hsmith/HSITS/1_Sequences/1_adapterclipped/FT1_${i}.gz \
/export/home/f-
k/hsmith/HSITS/1_Sequences/1_adapterclipped/fu1_${i}.gz \
/export/home/f-
k/hsmith/HSITS/1_Sequences/1_adapterclipped/RT2_${i}.gz \
/export/home/f-
k/hsmith/HSITS/1_Sequences/1_adapterclipped/ru2_${i}.gz \
ILLUMINACLIP:/export/home/f-k/hsmith/HSITS/Trimmomatic-0-
2.36/adapters/NexteraPE-PE.fa:2:30:10 LEADING:3 TRAILING:3
SLIDINGWINDOW:4:15 MINLEN:170


# paired end sequences
# adapters are already removed


pear \
-f /export/home/f-
k/hsmith/HSITS/1_Sequences/0_trimmed/FT1_${i}.gz \
-r /export/home/f-
k/hsmith/HSITS/1_Sequences/0_trimmed/RT2_${i}.gz \
-o /export/home/f-
k/hsmith/HSITS/1_Sequences/1_paired/${i}.fasta \
-n 150

# 150 is the minimum assembly length - any sequences below 150
bases are discarded


# convert fastq to fasta and corresponding qual files

convert_fastaqual_fastq.py \
-f /export/home/f-
k/hsmith/HSITS/1_Sequences/1_paired/${i}.fasta.assembled.fastq
\
-o /export/home/f-k/hsmith/HSITS/3_QIIME/fasta \
-c fastq_to_fastaqual


#add qiime labels using mapping file

add_qiime_labels.py \
-m /export/home/f-
k/hsmith/HSITS/3_QIIME/hs_mapping_file_final.txt \
-i /export/home/f-k/hsmith/HSITS/3_QIIME/fasta_checked \
-n 1000000 \
-c InputFileName \
```

```
-o /export/home/f-k/hsmith/HSITS/3_QIIME/checked_combined/

# count how many sequences there are in the combined file
count_seqs.py \
-i /export/home/f-
k/hsmith/HSITS/3_QIIME/checked_combined/combined_seqs.fna \
-o /export/home/f-
k/hsmith/HSITS/3_QIIME/checked_combined/combinedseqscount.txt

#Chimera check
# -compare combined sequences to chimera-free database
# -generate list of suspected chimeric sequences

identify_chimeric_seqs.py \
--chimera_detection_method usearch61 \
--input_fasta_fp /export/home/f-
k/hsmith/HSITS/3_QIIME/checked_combined/combined_seqs.fna \
--reference_seqs_fp /export/home/f-
k/hsmith/HSITS/3_QIIME/USearchChimera/arif_ITS2_DB.fasta \
--output_fp /export/home/f-
k/hsmith/HSITS/3_QIIME/USearchChimera3

#Delete chimeric sequences from combined sequence file

filter_fasta.py \
--input_fasta_fp /export/home/f-
k/hsmith/HSITS/3_QIIME/checked_combined/combined_seqs.fna \
--seq_id_fp /export/home/f-
k/hsmith/HSITS/3_QIIME/USearchChimera3/chimeras.txt \
--negate \
--output_fasta_fp /export/home/f-
k/hsmith/HSITS/3_QIIME/USearchChimera3/non_chimeric_seqs.fna

# count how many were excluded due to chimerism
count_seqs.py \
-i /export/home/f-
k/hsmith/HSITS/3_QIIME/USearchChimera3/non_chimeric_seqs.fna \
-o /export/home/f-
k/hsmith/HSITS/3_QIIME/USearchChimera3/nonchimericseqscount.tx
t

# pick OTUs using QIIME uclust method

pick_otus.py \
--otu_picking_method uclust_ref \
--similarity 0.99 \
--input_seqs_filepath /export/home/f-
k/hsmith/HSITS/3_QIIME/USearchChimera3/non_chimeric_seqs.fna \
--refseqs_fp /export/home/f-
k/hsmith/HSITS/3_QIIME/USearchChimera/arif_ITS2_DB.fasta \
--suppress_new_clusters \
```

```
--output_dir /export/home/f-
k/hsmith/HSITS/3_QIIME/uclust_ref_otus

#generate repset
pick_rep_set.py \
--input_file /export/home/f-
k/hsmith/HSITS/3_QIIME/uclust_ref_otus/non_chimeric_seqs_otus.
txt \
--fasta_file /export/home/f-
k/hsmith/HSITS/3_QIIME/USearchChimera3/non_chimeric_seqs.fna \
--result_fp /export/home/f-
k/hsmith/HSITS/3_QIIME/uclust_ref_otus/rep_set.fna

# no need to name taxa because names are in the Arif database
# make otu table

make_otu_table.py \
--otu_map_fp /export/home/f-
k/hsmith/HSITS/3_QIIME/uclust_ref_otus/non_chimeric_seqs_otus.
txt \
--taxonomy /export/home/f-
k/hsmith/HSITS/3_QIIME/USearchChimera/arif_ITS2_taxa.txt \
--output_biom_fp /export/home/f-
k/hsmith/HSITS/3_QIIME/ITS2_OTUs/its2_otu_table.biom

#Remove OTUs derived from sequences that comprise <0.05% of
all sequences.
#There is a high probability that such rare sequences are
spurious

filter_otus_from_otu_table.py \
--input_fp /export/home/f-
k/hsmith/HSITS/3_QIIME/ITS2_OTUs/its2_otu_table.biom \
--min_count_fraction 0.0005 \
--output_fp /export/home/f-
k/hsmith/HSITS/3_QIIME/ITS2_OTUs/otu_table_filtered.biom

# produce report of final OTU table

biom summarize-table \
--input-fp /export/home/f-
k/hsmith/HSITS/3_QIIME/ITS2_OTUs/otu_table_filtered.biom \
--output-fp /export/home/f-
k/hsmith/HSITS/3_QIIME/ITS2_OTUs/otu_table_summary.txt

# make taxonomic summary charts

summarize_taxa_through_plots.py \
--otu_table_fp /export/home/f-
k/hsmith/HSITS/3_QIIME/ITS2_OTUs/otu_table_filtered.biom \
```

```
--mapping_fp /export/home/f-
k/hsmith/HSITS/3_QIIME/hs_mapping_file_final.txt \
--parameter_fp /export/home/f-
k/hsmith/HSITS/3_QIIME/parameter_file.txt \
--mapping_category Bleached \
--sort --force \
--output_dir /export/home/f-
k/hsmith/HSITS/3_QIIME/ITS2_OTUs/its2_taxa_summary_bleach

# convert biom table output to excel readable format
biom convert -i table.biom -o table.from_biom_w_taxonomy.txt -
-table-type="OTU table" --to-tsv
```
